# Proteomics and bioinformatics analysis of cardiovascular related proteins in offspring exposed to gestational diabetes mellitus

**DOI:** 10.3389/fcvm.2022.1021112

**Published:** 2022-10-06

**Authors:** Hai-Tao Pan, Yi-Meng Xiong, Hong-Dan Zhu, Xiao-Liang Shi, Bin Yu, Hai-Gang Ding, Ren-Jie Xu, Jin-Long Ding, Tao Zhang, Juan Zhang

**Affiliations:** ^1^Shaoxing Maternity and Child Health Care Hospital, Shaoxing, China; ^2^The International Peace Maternity and Child Health Hospital, School of Medicine, Shanghai Jiao Tong University, Shanghai, China; ^3^Obstetrics and Gynecology Hospital of Shaoxing University, Shaoxing, China

**Keywords:** Gamete and Embryo-Fetal Origins of Adult Diseases, gestational diabetes mellitus (GDM), offspring, umbilical vessel, iTRAQ

## Abstract

**Introduction:**

Previous studies have demonstrated that exposed to the initial suboptimal intrauterine environment of gestational diabetes mellitus (GDM) may increase risk of cardiovascular disease in adulthood.

**Methods:**

In order to investigate the underlying mechanisms involved in the increased risk of cardiovascular diseases (CVDs) in the offspring of GDM, we applied a high-throughput proteomics approach to compare the proteomic expression profile of human umbilical vessels of normal and GDM offspring.

**Results:**

A total of significantly different 100 proteins were identified in umbilical vessels from GDM group compared with normal controls, among which 31 proteins were up-regulated, while 69 proteins were down-regulated. Differentially expressed proteins (DEPs) are validated using Western blotting analysis. The analysis of these differently expressed proteins (DEPs) related diseases and functions results, performed by Ingenuity Pathway Analysis (IPA) software. Based on “Diseases and Disorders” analysis, 17 proteins (ACTA2, ADAR, CBFB, DDAH1, FBN1, FGA, FGB, FGG, GLS, GSTM1, HBB, PGM3, PPP1R13L, S100A8, SLC12A4, TPP2, VCAN) were described to be associated with CVD, especially in Anemia, Thrombus and Myocardial infarction. Functional analysis indicated that DEPs involved in many cardiovascular functions, especially in “vasoconstriction of blood vessel” (related DEPs: ACTA2, DDAH1, FBN1, FGA, FGB, and FGG). Upstream regulator analyses of DEPs identifies STAT3 as inhibitor of ACTA2, FGA, FGB, and FGG.

**Conclusion:**

The results of this study indicate that intrauterine hyperglycemia is associated with an elevated risk of cardiovascular risk in the offspring.

## Introduction

A growing number of epidemiological and experimental studies suggest that exposure to adverse intrauterine environment during fetal development can be associated with chronic disease in later life, such as CVD, obesity, type 2 diabetes and cognitive disorder (1–6). The theory of Gamete and Embryo-Fetal Origins of Adult Diseases is used preferentially to describe these associations. Gestational diabetes mellitus (GDM) is defined as any degree of glucose intolerance with onset or first diagnosis during gestation. The prevalence of GDM ranged from 9.3 to 25.5% among 15 collaborating centers using the International Association of the Diabetes and Pregnancy Study Group (IADPSG) criteria (7). GDM is a serious health risk for both pregnant women and their offspring.

Emerging evidence suggests that the vasculature of women with a prior case of GDM is permanently altered, predisposing them to CVD. GDM also increases the offspring’s risk of developing hypertension and CVD. Elevated systolic blood pressure (SBP) and diastolic blood pressure (DBP) (4, 8–11), increased intima-media thickness (IMT) (12), increased cardiac septal hypertrophy (13), and vascular endothelial dysfunctions were observed in the offspring of GDM mother (14, 15). Consistent with epidemiological results, studies in animal models also showed that diabetes during pregnancy affected the development of fetal cardiovascular system (16, 17). In one of our previous works, we also have indicated that intrauterine hyperglycemia could induce IGT (impaired glucose tolerance) and abnormal blood insulin levels in both F1 and F2 offspring (18). In studies of the mechanisms of cardiovascular dysfunction caused by GDM, numbers of potential pathways have been implicated in endothelial cell, including reduced adenosine transport (14), impaired angiogenesis (15) and redox signaling (19). Although many previous studies have been conducted, its precise mechanism involved in the association between intrauterine hyperglycemia and a higher risk of cardiovascular anomalies has yet to be established.

In the present study, we aimed to investigate the cardiovascular risk proteins in offspring exposed to GDM. A proteomics analysis was conducted in umbilical vessels from newborns of mothers with GDM and normal controls using the isobaric tag for relative and absolute quantitation (iTRAQ)-labeling technique to compare the proteomic expression profile. We analyzed the related diseases and functions using ingenuity pathway analysis (IPA) software. The results of the present study may provide valuable information for further investigation of the mechanisms underlying the cardiovascular dysfunction induced by intrauterine hyperglycemia.

## Materials and methods

### Patients and umbilical cords

Umbilical cords from newborns of 25 mothers with mild GDM and 25 controls were collected by obstetricians in Shaoxing Maternity and Child Health Care Hospital, China. The Ethics Committee of Shaoxing Maternity and Child Health Care Hospital approved the study. All the participants enrolled in this study were with singleton pregnancy and ceased pregnancies with Cesarean section at full term. Tissue samples were stored snap frozen at −80^°^Cuntil use. The clinical characteristics of the proteomic participants included in this study are outlined in Table 1.

**TABLE 1:** Clinical characteristic of the participants.

Characteristics	Control	GDM	*P*-value
Cases	25	25	
Gestational age, wk	39.06 ± 0.52	38.85 ± 0.62	0.108
Birth weight, g	3439.57 ± 421.29	3468 ± 456.14	0.241
Maternal age, y	30.21 ± 3.51	31.42 ± 3.11	0.071
BMI of pre-pregnancy, kg/m^2^	22.12 ± 2.74	22.79 ± 4.12	0.058
Glycated hemoglobin, %	4.94 ± 0.27	5.10 ± 0.41	0.041*
Pregnancy weight gain, kg	15.65 ± 5.15	14.10 ± 8.59	0.187
OGTT 75 g, 0 h, mmol/L	4.47 ± 0.36	5.02 ± 0.68	< 0.001**
OGTT 75 g, 1 h, mmol/L	8.01 ± 0.93	10.92 ± 1.68	< 0.001**
OGTT 75 g, 2 h, mmol/L	6.56 ± 1.02	9.38 ± 1.60	< 0.001**
Placenta weight,g	598.35 ± 89.92	603.28 ± 116.46	0.097
TC in first-trimester, mmol/L	4.84 ± 0.87	4.78 ± 0.72	0.765
TG in first-trimester, mmol/L	1.23 ± 0.41	1.11 ± 0.14	0.108
HDL in first-trimester, mmol/L	1.67 ± 0.34	1.61 ± 1.48	0.772
LDL in first-trimester, mmol/L	3.12 ± 0.41	3.271 ± 0.37	0.692
Diagnosis	NS	GDM	
Presence of other major cardiovascular risk factor(s) in the mothers	None	None	

Data are showed as means ± SD, *P < 0.05, **P < 0.001 compared with control.

GDM were diagnosed between gestational weeks 24 and 28 after overnight fasting (for 8–12 h) by an oral glucose tolerance test (OGTT). According to the IADPSG diagnostic criteria, GDM was defined as fasting venous plasma glucose concentration ≥ 5.1 mM and ≥ 10.0 mM at 1-hand/or ≥ 8.5 mM at 2-h after drinking a solution with 75 g glucose. All women with GDM enrolled in the present study experienced dietary management without insulin treatment. Participants with maternal obesity factor (high pre-pregnancy BMI, excessive weight gain during pregnancy, abnormal lipid level in first-trimester) and macrosomia were excluded. The work described in the present study has been carried out in accordance with The Code of Ethics of the World Medical Association (Declaration of Helsinki). The study protocols were reviewed and approved by the Research and Ethics Committee of the Women’s Hospital, School of Medicine, Zhejiang University, China, and informed consents were provided by all participants.

### Isobaric tag for relative and absolute quantitation analysis

iTRAQ analysis was performed as previously described (20). Briefly, protein was extracted from umbilical artery and measured by BCA assay (Pierce, Rockford, IL, USA) according to the manufacturer’s protocol. Protein digestion was performed according to the FASP procedure, as described by Wisniewski et al. (21). Briefly, 200 μg of total-protein samples were diluted in 30 μL of solution including 4% SDS, 100 mM Tris-HCl pH 8.0 and 100 mM dithiotreitol, and were heated at 95^°^C for 5 min. After each sample was cooled to room temperature, the sample was loaded onto an ultrafiltration filter (cutoff 10 kDa, Sartorius, Goettingen, Germany). We added 200 μL UT buffer (8 M Urea and 150 mM Tris-HCl, pH 8.0) to the filter and centrifuged it at 14,000 g at 20^°^C for 30 min. Subsequently, 100 μL of iodoacetamide solution (50 mM iodoacetamide in UT buffer) was added for blocking reduced cysteines, and, the samples were incubated for 20 min in darkness. Then the filters were centrifuged at 14,000 g at 20^°^C for 20 min. The filters were washed with 100 μL UT buffer at 14,000 g for 20 min. This step was repeated 2 times. Then, 100 μL dissolution buffer (AB Sciex, Framingham, MA, USA) was added to the filter, and it was centrifuged at 14,000 g at 20^°^C for 30 min, and, this step was repeated twice. Finally, 40 μL of trypsin (Promega, Madison, WI, USA) buffer (2 μg trypsin in 40 μL dissolution buffer) were added, and, the samples were digested overnight at 37^°^C. Each filter unit was transferred to a new tube and centrifuged at 14,000 g at 20^°^C for 30 min. The concentration of resulting peptides was determined by UV light spectral density at OD_280_ (22).

The iTRAQ labeling of digested peptide samples was performed following the manufacturers protocol with 8-plex isobaric tags for relative and absolute quantitation (iTRAQ) labeling kit (AB Sciex, Framingham, MA, USA). Three umbilical arteries from the control group (C) were labeled with mass 114, 115 and 116 isobaric iTRAQ tags, the other three umbilical arteries from the GDM group were labeled with mass 117, 118, and 119 isobaric iTRAQ tags. Identical quantities of peptide mixtures from the 6 peptides mentioned above were labeled with reagent 113 and served as sample IS (internal standard). According to the manufacturers protocol, The labeling reactions were incubated for 2 h at room temperature before further analysis.

After iTRAQ-labeling the peptide samples were combined and subsequently purified using a strong cation exchange (SCX)-cartridge: Polysulfoethyl 4.6 × 100 mm column (5 μm, 200 Å, Poly LC Inc., Columbia, MD, USA). For LC-MS/MS analysis of the resulting peptides, we followed a previously described method (20). Protein identification and quantification for iTRAQ analysis data was carried out using the MASCOT search engine (version 2.2.1; Matrix Science, London, UK) embedded into Proteome Discoverer 1.3 (Thermo Electron, San Jose, CA, USA), searching against the Uniport database of human protein sequences (03-2013, 133549 entries, downloaded from: http://www.uniprot.org/) and the concatenated target-decoy database. The parameters were set as follows: Trypsin as digestion enzyme, cysteine carbamidomethylation as a fixed modification, Oxidation (M), Gln→Pyro-Glu (N-term Q), iTRAQ 8 plex (K), iTRAQ 8 plex (Y), and iTRAQ 8 plex (N-term) as the variable modification.

### Western blotting analysis

Human umbilical arteries were lysed in 1 × RIPA buffer with 1μg/mL leupeptin and 1μg/mL phenylmethylsulfonyl fluoride. Aliquots containing 30 μg of protein samples were separated by 12% SDS-PAGE and transferred electrophoretically to a nitrocellulose transfer membrane (Bio-Rad, Hercules, CA, USA). After blocked with 5% BSA in TBS containing 0.01% Tween 20 (TBST) for 1 h at room temperature, the membrane was incubated overnight at 4^°^C with primary antibodies against FGA (1:1,000, Abcam, Cambridge, MA, USA), ACTA2 (1:1,000, Abcam, Cambridge, MA, USA), IDH3A (1:1,000, Abcam, Cambridge, MA, USA), GAPDH (1:1,000, Abcam, Cambridge, MA, USA). Subsequently, membranes were washed 10 min for three times with TBST, then each membrane was incubated for 1 h at room temperature with the appropriate secondary antibody (anti-rabbit IgG, anti-mouse IgG; 1:5,000; Abcam). The protein intensities were determined and analyzed using Odyssey^®^ Imager (LI-COR Biosciences, Lincoln, NE, USA).

### Bioinformatics analysis of differentially expressed proteins

Proteins exhibiting at least a ± 20% fold change in expression were determined as significantly different. The Cluster 3.0^1^ and Java Tree view software^2^ were further used to evaluate the capability of the resulting feature proteins in differentiating the two groups of samples. IPA software (QIAGEN, Redwood 185 City, CA) was employed for functional analysis of the identified DEPs between the two groups. In the functional network analysis, the DEPs were represented as nodes, and the biological relationship between two proteins was represented as an edge (line), which was supported by the published articles or the canonical information embedded in the IPA database. Downstream biological processes analysis, which was used to predict the downstream effects of the DEPs based on the observed gene expression changes, was also on the strength of the canonical information embedded in the IPA database. The calculated z-score can be used to infer the activation states (“increased” or “decreased”) of implicated biological processes. Fisher’s exact test was used to calculate a *p*-value to determine the probability that the association between proteins in the dataset, and the biological process could be explained by chance alone.

### Statistical analysis

All analysis was performed with the software Statistical Analysis Software (SPSS 17.0 software, SPSS Inc., USA). Data are displayed as means ± SEM. Statistical evaluation was conducted using an unpaired Student’s *t*-test, and *P* < 0.05 was considered statistically significant.

## Results

### The clinical characteristics of the participants

Twenty five pregnancies with GDM and 25 normal controls were enrolled in the present study, and the specific clinical characteristics of the participants and delivery data were shown in Table 1. None of them experienced major cardiovascular risk factors, such as cardiovascular system diseases, history of type 2 diabetes, hyperthyroidism, assisted reproductive technology treatment, smoking, congenital defect, and any other pregnancy complications. There was no significant difference between GDM group and the controls about the maternal age, gestational weeks, pre-pregnancy BMI, weight gain during pregnancy, lipid level in first-trimester, birth weight and gender distribution of newborns (all *P* > 0.05). The serum glucose concentration at 0,1, and 2 h of OGTT in the GDM group were significantly higher than that in the normal pregnancies (*p* < 0.001, respectively). Three pairs of the collected umbilical vessels were selected to compare the proteome profiles between GDM and controls by iTRAQ-LC-MS/MS.

### Overview of the proteomics analysis

**FIGURE 1:**
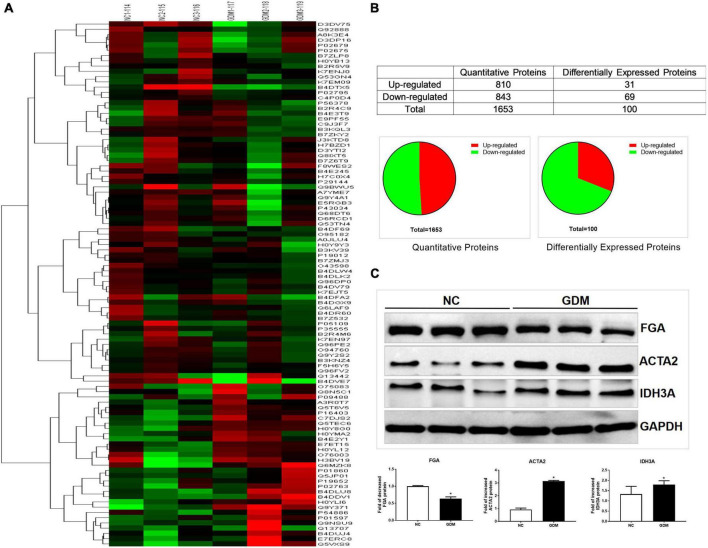
iTRAQ analysis results for the GDM umbilical vessels. **(A)** Hierarchical clustering of DEPs. **(B)** The statistic of the proteins identified in umbilical vessels. **(C)** Validation of the differential expression of the selected proteins in umbilical vessels by western blotting: FGA, ACTA2, IDH3A. Data are presented as mean ± SE (*n* = 3). *p < 0.01.

To identify the differentially expressed proteins (DEPs) in the umbilical arteries between GDM and controls in genome-wide level, iTRAQ-LC-MS/MS was performed in 3 GDM samples and 3 control samples. In the present study, a total of 1,653 proteins were quantified in the umbilical arteries from both GDM and the controls (Supplementary Table 1). The mass spectrometry proteomics data have been deposited to the ProteomeXchange Consortium (http://proteomecentral.proteomexchange.org) via the iProX partner repository (23) with the dataset identifier PXD024892. Using the screening criteria of fold change over ± 1.2, 100 proteins were regarded as differentially expressed in umbilical arteries from GDM group, of which, 31 proteins were up-regulated and 69 proteins were down-regulated (Supplementary Table 2 and Figure 1B). Hierarchical clustering analysis was further performed with the identified 100 DEPs mentioned above. After unsupervised clustering, the GDM group were significantly distinguished from the controls (Figure 1A), indicating the significant effect of intrauterine hyperglycemia on the expression of proteins in umbilical vessels of newborn.

### Western blotting validation

To validate the results carried out by iTRAQ-LC-MS/MS, Western blotting was conducted in additional three umbilical vessels from GDM and three normal umbilical vessels. Based on the significant expression difference and the biological function in the cardiovascular system, three proteins, including FGA, ACTA2, and IDH3A, were selected for further investigation. As shown in Figure 1C, the change trends of these three proteins within Western blotting analysis were in accordance with iTRAQ analysis results.

### Bioinformatics analysis of differentially expressed proteins

**FIGURE 2:**
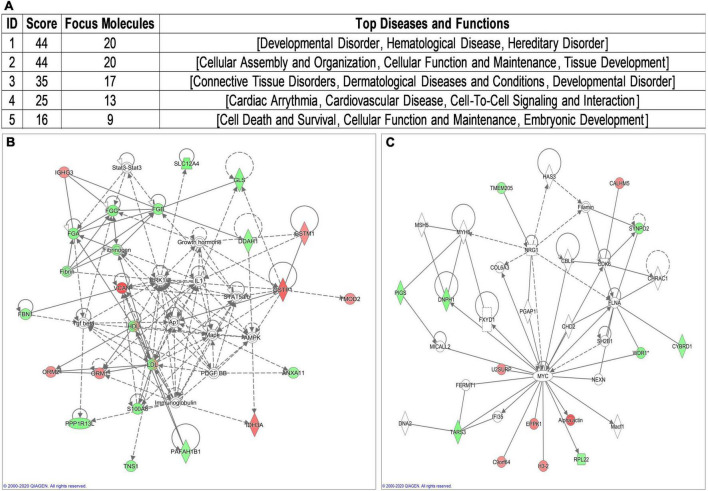
Visual representation of the principal network generated by ingenuity pathway analysis (IPA). **(A)** Principal network generated by IPA. **(B)** Developmental Disorder associated network. **(C)** Cardiovascular Disease associated network.

To assist functional interpretation of the DEPs, the bioinformatics analysis was performed based on the 100 DEPs. These genes were loaded into IPA database (IPA^®^, QIAGEN)^3^ for pathway, disease and function, and network analysis. Three cardiovascular signaling pathways identified by IPA software (Supplementary Table 3 and Supplementary Figure 1) including “Atherosclerosis Signaling” (related gene: ORM1, ORM2, and S100A8). According to overlapping *p*-values, these 73 subcategories showed in “Disease and Disorder” analysis, including “Developmental Disorder” and “CVD” (Supplementary Table 4 and Supplementary Figure 2). In addition to the gene ontology enrichment, 22 subcategories involed in “Physiological System Development and Function,” especially in “Cardiovascular System Development and Function” (Supplementary Table 5 and Supplementary Figure 3). Network analysis identified five biological networks (Figure 2A), which included developmental network (Figure 2B) and cardiovascular network (Figure 2C). The most related network emerged comprising 20 of those DEPs. It is associated with the IPA functions “Developmental Disorder” (Figure 2B).

### Cardiovascular risk proteins analysis

**FIGURE 3:**
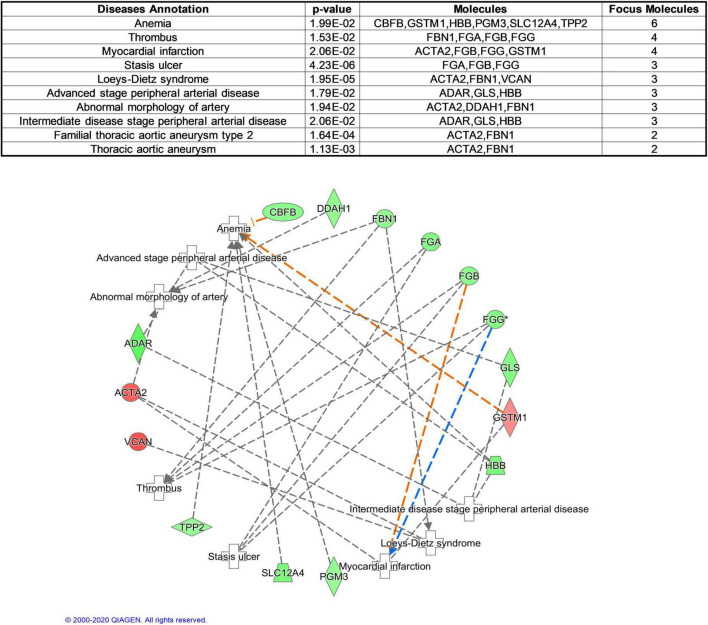
Cardiovascular Disease analysis of differentially expressed proteins between normal and GDM umbilical vessels. For this developmental disease network, genes or gene products are represented as *nodes*, and the biological relationship between two nodes is represented as an *edge*. All edges are supported by at least one publication as stored in the Ingenuity Knowledge database. The intensity of the node color indicates the degree of up- (red) or down- (green) regulation.

**FIGURE 4:**
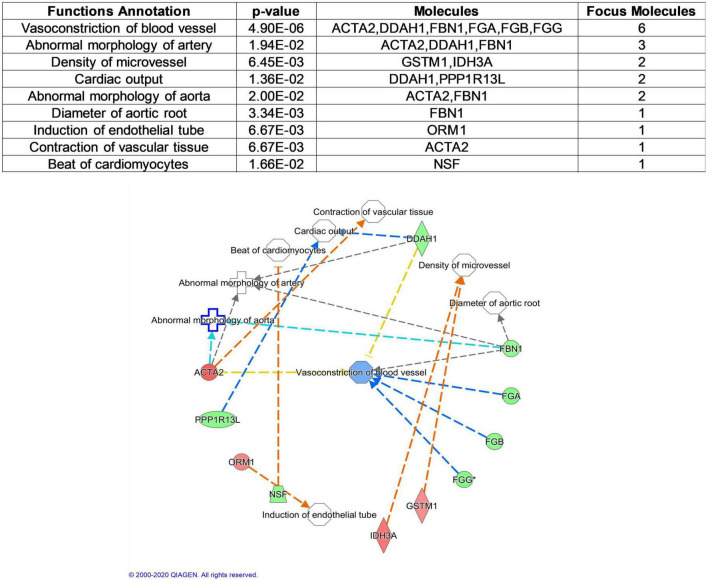
Cardiovascular Functional analysis of differentially expressed proteins between normal and GDM umbilical vessels. For this cardiovascular function network, genes or gene products are represented as *nodes*, and the biological relationship between two nodes is represented as an *edge*. All edges are supported by at least one publication as stored in the Ingenuity Knowledge database. The intensity of the node color indicates the degree of up- (red) or down- (green) regulation.

To better understand cardiovascular risk proteins in offspring exposed to GDM, these DEPs were further analyzed in CVDs and functions. Based on “Diseases and Disorders” analysis, 17 proteins (ACTA2, ADAR, CBFB, DDAH1, FBN1, FGA, FGB, FGG, GLS, GSTM1, HBB, PGM3, PPP1R13L, S100A8, SLC12A4, TPP2, VCAN) were described to be associated with CVD, especially in Anemia, Thrombus and Myocardial infarction (Figure 3). Based on “Physiological System Development and Functions” analysis, 11 proteins (ACTA2, DDAH1, FBN1, FGA, FGB, FGG, GSTM1, IDH3A, NSF, ORM1, PPP1R13L) were described to be associated with Cardiovascular System Development and Function, especially in the function of “vasoconstriction of blood vessel” (Figure 4).

**FIGURE 5:**
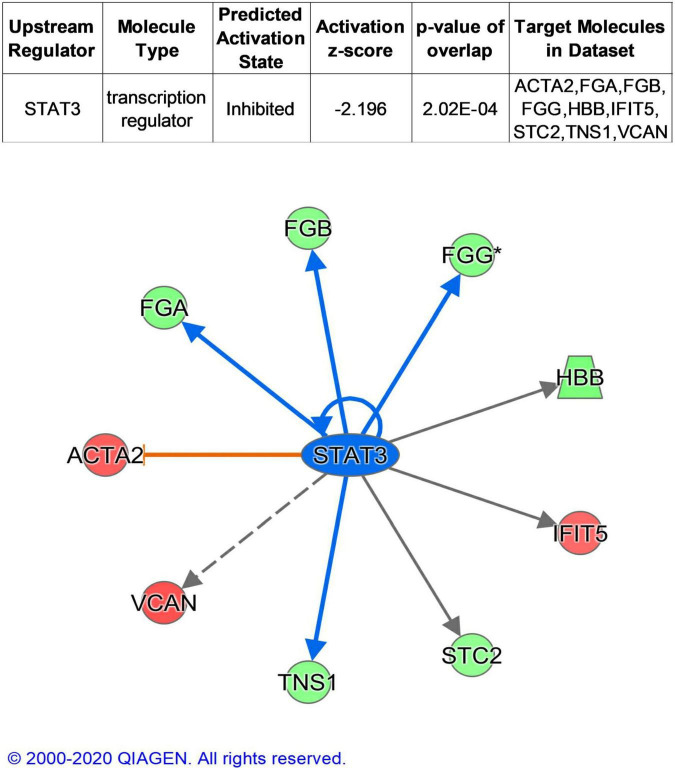
Upstream analysis of DEPs. Colorized nodes represented our input proteins. Green, down-regulated proteins. Red, up-regulated proteins.

The term “upstream regulator” as used in IPA refers to any molecule that can affect the expression of another molecule. In upstream regulator analysis, STAT3 was predicted in inhibited state (z-score = −2.196) and regulated ACTA2, FBN1, FGA, FGB, and FGG (Figure 5).

## Discussion

Cardiovascular and metabolic disorders often present in adult life, but may have their origins in changes to the intrauterine environment during fetal development. Previous studies including epidemiological investigations and experimental projects have demonstrated the alteration of vascular function and cardiovascular system in the offspring of GDM (8, 24). The current study firstly focus on the proteomics of umbilical vessels from GDM and normal controls, of normal birth weights, which excluded maternal obesity factors (high pre-pregnancy BMI, excessive weight gain during pregnancy, abnormal lipid level in first-trimester). Our results demonstrated that the intrauterine hyperglycemia indeed individually affected the expression patterns of proteins and vessel function.

Several studies implicated that the association between maternal hyperglycemia and childhood metabolic outcomes was attenuated after adjusting for covariates including maternal BMI and weight gain during pregnancy (11), overall, existing evidence suggested gestational diabetes as an independent element. To date, intensified treatment during pregnancy of maternal hyperglycemia reduce maternal weight gain and macrosomia at birth, whether similar associations still exist between maternal hyperglycemia and offspring cardiovascular outcomes is few studied. In the current study, participants with no maternal obesity factors (high pre-pregnancy BMI, excessive weight gain during pregnancy, abnormal lipid level in first-trimester) were recruited, and macrosomia was excluded.

The physiological and pathological regulation of cardiovascular function derives mainly from the collaboration between vascular endothelial cells and VSMCs (Vascular smooth muscle cells). Previous studies stated that characteristics and alterations of umbilical vessels could offer valuable information since umbilical vessel cells experienced the effect of same intrauterine environment (20, 25–27). In consideration of the ethics limited, umbilical vessels is thought to be the best tissue that could be collected in clinical.

Based on the identified DEPs in umbilical arteries between GDM and normal controls, our results suggested that the “Cardiovascular System Development and Function” was impaired exposure to intrauterine hyperglycemia. In this content, the top related function was “Vasoconstriction of blood vessel,” this is consistent with a large number of epidemiological results. These suppressed physiological processes in the umbilical vessels from GDM might lead to impaired vascular repair under stress or diabetic vessel pathological damage.

In this study, we found 20 DEPs (ACTA2, ADAR, CBFB, DDAH1, FBN1, FGA, FGB, FGG, GLS, GSTM1, HBB, PGM3, PPP1R13L, S100A8, SLC12A4, TPP2, VCAN, IDH3A, NSF, ORM1) related with Cardiovascular system. ACTA2 gene mutations in adults associated with thoracic aortic aneurysm and dissection (TAAD) (28). Transgenetic mouse model study proposes that sufficient ADAR2 enzyme activity might play a vital role in preventing cardiovascular defects (29). CBFB may impair the primitive hematopoiesis (30). FBN1 is a gene with a well characterized role in the pathogenesis of thoracic aortic aneurysm (TAA) in the context of Marfan syndrome (31). Inferring that variation in genomic sequences that regulate the fibrinogen genes (FGA, FGB, and FGG) may affect hepatic fibrinogen production and perhaps CVD risk (32). MicroRNA-200c exacerbates the ischemia/reperfusion injury of heart through targeting the glutaminase (GLS)-mediated glutamine metabolism (33). Combined GSTM1*0/GSTA1*A genotypes might be considered as genetic markers for cardiovascular death risk in ESRD patients, which may permit targeting of preventive and early intervention (34). HBB identified to be related to Abdominal aortic aneurysm (AAA) (35). PPP1R13L affecting NFkB activity may be candidate genes in the study of human CVD (36). Levels of S100A8/A9, a proinflammatory and prothrombotic protein complex, are increased in several diseases, and high levels predispose to CVD (37). Total absence of the VCAN gene halts heart development at a stage prior to the heart’s pulmonary/aortic outlet segment growth (38).

In summary, in the present study, we discovered that the expression profile of proteins in umbilical vessels of newborns from GDM group was different from that in normal controls. The bio-informatics analysis suggested that some DEPs might play important roles in cardiovascular dysfunction of GDM children. Our findings would contribute to the exploration of the potential mechanism underlying the dysregulated balance of apoptosis and autophagy in vessels, angiogenesis and endothelial cell dysfunction in the offspring of GDM pregnancies. However, deeper analyses will still mostly need to be performed to explore the key factors and potential preventive and therapeutic strategies of cardiovascular dysfunction in GDM offspring.

## Data availability statement

The datasets presented in this study can be found in online repositories. The names of the repository/repositories and accession number(s) can be found in the article/Supplementary material.

## Ethics statement

The studies involving human participants were reviewed and approved by the Ethics Committee of Shaoxing Maternity and Child Health Care Hospital. The patients/participants provided their written informed consent to participate in this study.

## Author contributions

H-TP and Y-MX: writing—original draft. H-DZ: methodology. X-LS: formal analysis. BY: visualization. H-GD: validation. R-JX and J-LD: data curation. JZ and TZ: project administration. All authors contributed to the article and approved the submitted version.

## Footnotes

1
http://bonsai.hgc.jp/~mdehoon/software/cluster/software.htm

2
http://jtreeview.sourceforge.net

3
www.qiagen.com/ingenuity

## Supplementary material

Supplementary Figure 1 Cardiovascular related pathways.

Supplementary Figure 2 Disease and disorder analysis.

Supplementary Figure 3 Functional analysis.

Supplementary Table 1 A total of 1,653 proteins quantified in the umbilical arteries from both GDM and the controls.

Supplementary Table 2 A total of 100 differentially expressed proteins (DEPs) in umbilical vessels.

Supplementary Table 3 Ingenuity canonical pathways analysis.

Supplementary Table 4 Disease and disorder analysis.

Supplementary Table 5 Functional analysis.

## Abbreviations

GDM: Gestational Diabetes Mellitus
DEPs: Differentially Expressed Proteins
FDR: False Discovery Rate
IPA: Ingenuity Pathway Analysis
SCX: Strong Cationic-exchange Chromatography
SBP: Systolic Blood Pressure
IMT: Intima-Media Thickness
FGA: Fibrinogen Alpha Chain
ACTA2, Actin: aortic smooth muscle
IDH3A: Isocitrate dehydrogenase (NAD) subunit alphamitochondrial.
