# Supplementary figures and images for: Proteomics and bioinformatics analysis of cardiovascular related proteins in offspring exposed to gestational diabetes mellitus
Source: Front Cardiovasc Med. 2022 Oct 6;9:1021112. doi: 10.3389/fcvm.2022.1021112 (PMC9582427; doi:10.3389/fcvm.2022.1021112)

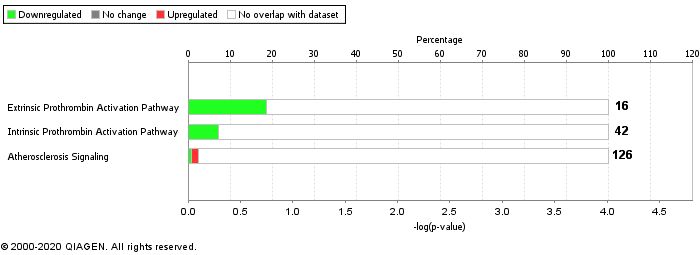

Supplement: Supplementary Figure 1 — Cardiovascular related pathways. [file Image_1.JPEG]

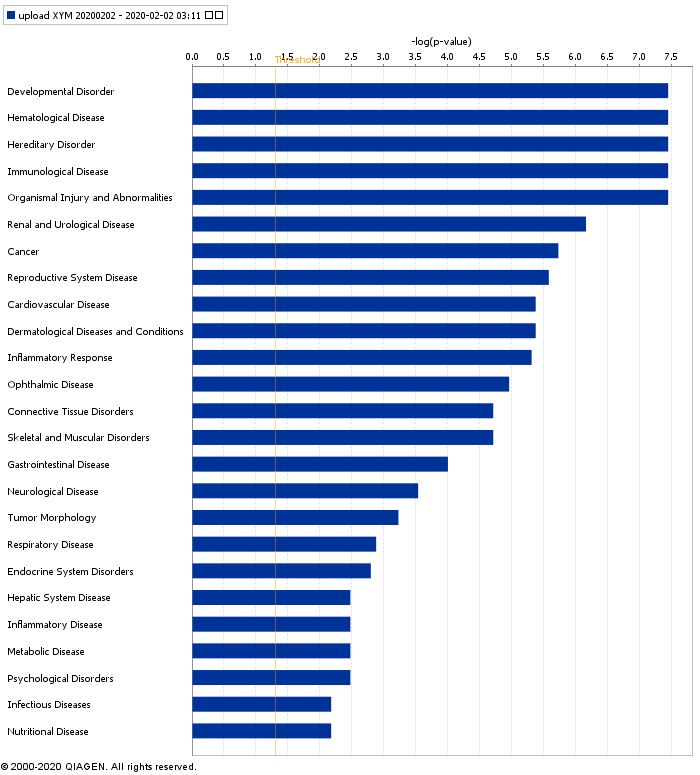

Supplement: Supplementary Figure 2 — Disease and disorder analysis. [file Image_2.JPEG]

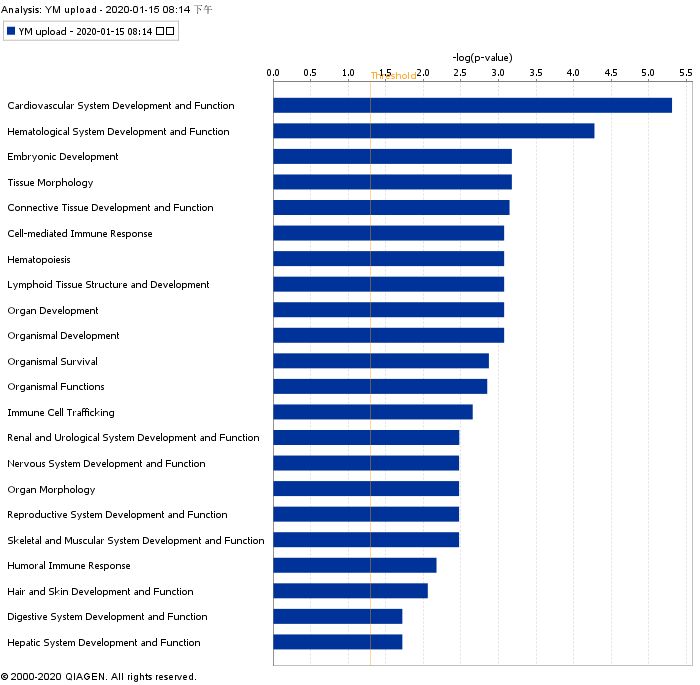

Supplement: Supplementary Figure 3 — Functional analysis. [file Image_3.JPEG]
